# The Brief Symptom Inventory-9 (BSI-9): Development and validation in a German general population sample

**DOI:** 10.1186/s40359-024-01890-8

**Published:** 2024-07-30

**Authors:** C. MacDonald, K. Brophy, A. Coroiu, Elmar Braehler, A. Körner

**Affiliations:** 1 McGill University, Montreal, QC Canada; 2 Nicotine Dependence Service, Center for Addiction and Mental Health (CAMH), Toronto, ON Canada; 3 Department of Medical Psychology and Medical Sociology, Medical Center of the University of Leipzig & Department of Psychosomatic Medicine and Psychotherapy, Medical Center of the University of Mainz, Leipzig, Germany

## Abstract

**Background:**

The Brief Symptom Inventory-18 (BSI-18) is a self-report questionnaire with three subscales, somatisation, anxiety, and depression, based on longer measures of distress. The present study proposes a shorter, nine-item version (BSI-9) of the BSI-18 as a brief screening tool for distress.

**Methods:**

Confirmatory factor analyses and reliability and validity analyses were carried out using a representative sample of the German general population. Confirmatory factor analysis demonstrates a good model fit for the three-dimensional BSI-9.

**Results:**

The total scale was found to have strong internal consistency (α_Cronbach_ = 0.87 for the global severity index). The internal consistency coefficients of the three-item subscales reflect the brevity of these scales (somatisation ﻿α_Cronbach_ = 0.72, depression ﻿α _Cronbach_ = 0.79, anxiety ﻿α_Cronbach_ = 0.68). The subscales were found to be significantly related with subscales of the Patient Health Questionnaire-4 and Hopkins Symptom Checklist-25.

**Limitations:**

The present study used a limited number of distress measures, and a more recent dataset would be useful to provide a more current picture of the general population’s distress levels.

**Conclusions:**

The BSI-9 provides a short, valid, and reliable screener for distress in the general population. Future work should examine its utility in clinical settings and different cultural contexts.

The Brief Symptom Inventory – 18 items (BSI-18; [1]) is a self-report questionnaire used to assess distress in both clinical and general populations [2–5]. It is the shortest version of a lineage of distress measures that began with the Symptom Checklist-90-Revised (SCL-90-R; [6]), a 90-item self-report measure of distress. The SCL-90-R assesses nine-symptom dimensions, (i.e. somatisation, obsessive-compulsive, interpersonal sensitivity, depression, anxiety, hostility, phobic anxiety, paranoid ideation, psychoticism, and a global score) and has been validated for use in both clinical and community samples [7]. The SCL-90-R was shortened into the Brief Symptom Inventory (BSI; [6]), which includes 53 items, maintaining the nine symptom dimensions of the SCL-90-R. The BSI-18 was developed as an even briefer measure of distress and is commonly modeled using a three-factor structure, i.e., anxiety, depression, somatisation, a one-factor structure, i.e., the global severity index [8, 9], or a four-factor structure in which the anxiety facet is divided into panic and agitation [1, 10]. Reducing the number of factors from nine to three improved the structural validity of the scale, as the three remaining factors were more homogenously related to distress [10], required less time for administration [11], allowed for a reduced clinical burden on both clinicians and patients [11, 12], and provided the most current assessment of distress symptoms [13].

The BSI-18 has been validated for use in several clinical populations, including patients diagnosed with traumatic brain injury, Parkinson’s disease patients, organ transplantations, and cancer patients [3, 9, 14, 15], as well as the general population [2, 16]. The BSI-18 has been translated into multiple languages and validated in numerous cultural and linguistic settings [9, 16, 17].

Researchers continue to examine and refine the measurement of psychological distress, including shortening measures to improve utility and efficiency in clinical practice [12]. Reducing the number of items in a measure, while retaining strong psychometric properties, facilitates more efficient and effective research, decreases the burden on patients as well as clinicians by reducing the time required to complete, score, and interpret these measures.

## Study objective and hypotheses

The aims of the current study are: (1) to develop a brief, nine-item German version of the BSIand (2) to assess the psychometric properties of the new BSI-9. We predicted that the BSI-9 would show positive correlations with both the Patient Health Questionnaire-4 and the Hopkins Symptom Checklist-25, other screening measures of distress. Specifically, we expected large correlations between corresponding subscales (i.e. between anxiety subscales and between depression subscales) of the BSI-9 and of the other measures of distress, compared to smaller correlations between non-corresponding subscales (e.g., between the somatisation subscale and subscales capturing anxiety or depression).

## Method

### Participants and Procedures

Data from a representative sample of the German population (*N* = 2520) were collected in 2009 by a demography consulting company from Germany (USUMA, Berlin). Representativeness was ensured by age, gender, and education distribution, according to the Federal Statistical Office (Franke et al., 2017). Participants were selected using a random-route procedure, which randomly selects households and household members. The current study reports on participants who provided complete responses for the target measure, the BSI-18, resulting in a sample size of *N* = 2482.

### Measures

#### The Brief Symptom Inventory 18 (BSI-18)

The BSI-18 is an 18-item self-report measure of distress [18]comprised of three scales, somatization, anxiety, and depression. Participants are asked to rate how often each item distressed them in the past seven days, with Likert-type responses ranging from 1 to 5, where 1 = *not at all*; 2 = *a little bit*; 3 = *moderately*; 4 = *quite a bit*; 5 = *extremely*. The German version used in this study has been validated for use in the German general population [16]. Factorial studies of the German BSI-18 support the use of three subscales as well as the use of a global index, i.e., the sum score across all items [16]. Internal consistency reliability (Cronbach’s alpha) was 0.84 for anxiety, 0.87 for depression, 0.82 for the somatization subscales and 0.93 for the global severity index [16]. In terms of convergent validity of the German version, corresponding anxiety and depression scales of the BSI-18 and Patient Health Questionnaire-4 [19], i.e., anxiety and depression subscales, were more strongly correlated than non-corresponding subscales, i.e., the somatisation subscale of the BSI-18 [16]. Other language versions of the BSI-18 have been shown to have good convergent validity, sensitivity, and specificity [20–22].

#### The Patient Health Questionnaire-4 (PHQ-4)

The PHQ-4 is a 4-item self-report questionnaire that consists of a two-item anxiety scale (GAD-2) and a two-item depression scale (PHQ-2) with a Likert-type response scale assessing symptom severity. It has been validated for use in the general population [19, 23]. Participants are asked to rate their symptoms during the last two weeks as being present 0 = *not at all*, 1 = *several days*, 2 = *more than half the days*, and 3 = *nearly every day*. The internal consistency was found to be 0.78 for the depression subscale and 0.85 for the anxiety subscale, while the internal consistency for the total scale was 0.82 [23].

#### The Hopkins Symptom Checklist-25 (HSCL-25)

The HSCL-25 is a 25-item self-report measure that consists of two subscales measuring anxious and depressive symptoms. The response options range from 1 to 4, with 1 = *not at all*, 2 = *a little*, 3 = *quite a bit*, and 4 = *extremely*. Mean sum scores can be calculated for the anxiety (10 items) and depression (15 items) subscales. A global score can be derived by computing the mean across all 25 items. The German version of the HSCL-25 was validated by [24] in the German general population. The internal consistency was 0.84 for the anxiety subscale, 0.92 for the depression subscale, and 0.94 for the total score [24].

### Statistical analyses

The total sample (*N* = 2482) was split randomly into two datasets: A (*n* = 1255) and B (*n* = 1227). Descriptive statistics (%, mean, standard deviation, range) of demographic characteristics (Table 1) as well as means and standard deviations for all measures (Table 2) were computed for both subsamples using SPSS for Mac OSX v24. Dataset A was used to examine the factor structure of the German BSI-18, previously reported in [16] and to select the items for the briefer German BSI-9. Dataset B was used to investigate the factorial structure and other psychometric properties of the German BSI-9.

**Table 1:** Sample demographic information separated by statistical analysis group

	Group A (*N* = 1,255)	Group B (*N* = 1,227)
Age (years)		
*M* (SD)	50.13 (18.56)	50.76 (18.62)
Range	14–93	14–91
	*N* (%)	*N* (%)
Gender		
Female	675 (53.78)	662 (53.95)
Male	580 (46.22)	565 (46.05)
Relationship status		
Married	631 (50.28)	631 (51.43)
Married (separated)	15 (1.20)	16 (1.30)
Single	297 (23.67)	287 (23.39)
Divorced	147 (11.71)	131 (10.68)
Widowed	165 (13.15)	162 (13.20)
Education level		
Currently enrolled in secondary school	54 (4.30)	49 (3.99)
Basic education (≤ 9 years)	563 (44.86)	577 (47.03)
Secondary school	424 (33.78)	411 (33.50)
Technical college or university entrance qualification	130 (10.36)	112 (9.13)
Four or more years of university	84 (6.69)	78 (6.36)
Household net income/month (Euro)		
< 1000	135 (10.76)	141 (11.49)
1000 to < 2500	784 (62.67)	735 (59.90)
2500 to < 3500	203 (16.18)	215 (17.52)
3500 to < 5000	74 (5.90)	86 (7.00)
> 5000	20 (1.59)	17 (1.39)
Missing	39 (3.1)	33 (2.69)

**Table 2:** Pearson correlation coefficients (subsample B)

	1	2	3	4	5	6	7	8	9	*N*	M (SD)
1) BSI-9 Tot	1									1,255	0.26 (0.40)
2) BSI-9 Som	0.81	1								1,255	0.24 (0.43)
3) BSI-9 Dep	0.90	0.56	1							1,255	0.28 (0.51)
4) BSI-9 Anx	0.89	0.60	0.73	1						1,255	0.24 (0.41)
5) HSCL-25 Tot	0.79	0.62	0.73	0.71	1					1,226	1.29 (0.35)
6) HSCL-25 Dep	0.78	0.57	0.75	0.67	0.97	1				1,226	1.29 (0.39)
7) HSCL-25 Anx	0.70	0.60	0.58	0.66	0.90	0.77	1			1,226	1.29 (0.34)
8) PHQ-4 Tot	0.77	0.49	0.77	0.71	0.69	0.69	0.59	1		1,222	0.37 (0.53)
9) PHQ-4 Dep	0.71	0.48	0.71	0.62	0.62	0.63	0.51	0.93	1	1,222	0.40 (0.59)
10) PHQ-4 Anx	0.72	0.43	0.71	0.70	0.66	0.65	0.58	0.92	0.71	1,222	0.33 (0.57)

Note. *p* < .01 for all analyses reported; (1) BSI-9 Total = Brief Symptom Inventory, 9-item, total score; (2) BSI-9 Som = BSI-9 Somatization Subscale; (3) BSI-9 Dep = BSI-9 Depression Subscale; (4) BSI-9 Anx = BSI-9 Anxiety Subscale; (5) HSCL-25 Total = Hopkins Symptom Checklist-25, 25-item, total score; (6) HSCL-25 Dep = HSCL-25 Depression Subscale; (7) HSCL-25 Anx = HSCL-25 Anxiety Subscale; (8) PHQ-4 Total = Patient Health Questionnaire, 4-item, total score; (9) PHQ-4 Dep = PHQ-4 Depression Subscale; (10) PHQ-4 Anx = PHQ-4 Anxiety Subscale

#### Dataset A: Confirmatory Factor Analysis (CFA) with BSI-18

Based on previous reports on the factor structure of the German BSI-18 (Franke et al., 2017), two models were fitted using CFA in MPlus v.8 [25]: a one-factor model, including all 18 items, and a three-factor model corresponding to the three subscales of the BSI-18: anxiety, depression, somatization. Item responses were ordinal Likert data and were therefore modeled using the weighted least squares (WLSMV) estimator. In line with recommended guidelines for assessing model fit [26], in addition to the chi-square test, which is highly sensitive to sample size and can lead to inaccurate rejection of the model fit [27], we used a combination of the following model fit indices: the Comparative Fit Index (CFI; [28]), the Tucker-Lewis Index (TLI; [29]), the Root Mean Square Error of Approximation (RMSEA; [30]), and the Standardized Root Mean Square Residual (SRMR; [31]). The model fit was considered acceptable if $\:\chi\:$^2^*p* > 0.05, TLI $\:\ge\:$0.95; CFI $\:\ge\:$ 0.90, RMSEA <0.08; and SRMR <0.08, AVE<0.5 [32].

#### Dataset A: item selection for the BSI-9

The nine items for the BSI-9 were selected based on examination of the factor loadings for the BSI-18 items, examination of modification indices, and theoretical support for symptoms belonging to each subscale.

#### Dataset B: CFA with BSI-9

Once relevant items were selected for the BSI-9, the same two CFA models tested with the BSI-18 (but fewer items for the three-factor model) were also tested with the BSI-9 using the same modelling methods.

#### Dataset B: reliability and validity of the BSI-9

Cronbach’s alpha was computed for the BSI-9 total and subscales. Convergent and divergent validity of the BSI-9 were assessed through Pearson correlations with the corresponding subscales of the PHQ-4 and HSCL-25. Cohen’s effect sizes were used to assess the magnitude of the bivariate correlations, with *r* ≤ .10 indicating small, *r* = .30 indicating moderate, and *r* = .50 indicating large correlations [33].

## Results

### Sample demographic information

The demographic characteristics for both datasets are presented in Table 1.

### Development of the BSI-9

#### CFA with the BSI-18

A one-factor CFA with the BSI-18 showed an acceptable fit, $\:\chi\:$^*2*^(df = 135, *n* = 1,255) = 1133.39, *p* < .001; RMSEA = 0.08, 95% Confidence Interval (CI) [0.07-0.08]; TLI = 0.94; CFI = 0.95; SRMR = 0.06. A three-factor model also showed an acceptable fit $\:\chi\:$^2^(df = 132, *n* = 1,255) = 576.26, *p* < .001; *RMSEA =* 0.05, 95% CI [0.05-0.06]; *TLI* = 0.97; *CFI* = 0.98; *SRMR* = 0.04).

#### Selection of the items for the short version of BSI, the BSI-9

First, the item pool was narrowed to 12 items, four items per subscale, based on the items with the highest factor loadings (Table 3). Next, theoretical background (i.e., construct definitions) and modification indices were considered to shorten the scale to nine items, three items per subscale. Specifically, modification indices were used to narrow an initial set of items that correlated most strongly with theory. Items with larger modification indices were retained for the final scale. The minimum number of items (i.e., three) was selected for each subscale was chosen to prioritize the brevity of the scale [34].

**Table 3:** Descriptive statistics and factor loadings for the BSI-9

Item	M (SD)	Factor Loading (SE)	95% Confidence Interval
*Somatisation*	.*28* (0.49)		
4. Pains in heart or chest	*0.23* (0.56)	0.76 (0.03)	0.70-0.82
7. Nausea or upset stomach	*0.27* (0.61)	0.77 (0.03)	0.71-0.83
16. Feeling weak in parts of your body	.*33* (0.68)	0.85 (0.02)	0.81-0.89
*Depression*	*0.33* (0.60)		
2. Feeling no interest in things	.*34* (0.66)	0.81 (0.02)	0.77-0.85
8. Feeling blue	.*26* (0.65)	0.93 (0.01)	0.91-0.95
14. Feeling hopeless about the future	*0.38* (0.83)	0.82 (0.02)	0.78-0.86
*Anxiety*	*0.32* (0.50)		
3. Nervousness or shakiness inside	*0.27* (0.60)	0.83 (0.02)	0.79-0.87
6. Feeling tense or keyed up	*0.52* (0.77)	0.73 (0.02)	0.69-0.77
18. Feeling fearful	.*19* (0.53)	0.78 (0.03)	0.72-0.84

Note. M = Mean; S.D. = Standard Deviation; S.E. = Standard Error

#### CFA with the BSI-9

A one-factor CFA in subsample B showed an acceptable model fit ($\:\chi\:$^*2*^(df = 28, *n* = 1227) = 512.41, *p* < .001; RMSEA = 0.12, 95% CI [0.11-0.13]; TLI = 0.92; CFI = 0.94; SRMR = 0.06). A three-factor CFA showed an adequate fit ($\:{\chi\:}$^2^(24) = 80.78, *p* < .001; RMSEA = 0.04 [0.03-0.06]; TLI = 0.99; CFI = 0.99; SRMR = 0.02). Factor loadings and 95% Confidence Intervals for the BSI-9 items can be found in Table 3.

#### Reliability and convergent validity of the BSI-9

McDonald’s omega was 0.73 for the somatisation subscale, 0.80 for the depression subscale, 0.69 for the anxiety subscale, and 0.84 for the global severity index. The correlations between the subscales of the BSI-9 and, the PHQ-4 and HSCL-25 were moderate to large in magnitude and significant at the 0.01 level (Table 2).

## Discussion

To date, the BSI-9 is the shortest version of the Brief Symptom Inventory, a widely used measure of distress in both clinical and general populations. In the current analyses, the one and three factor structures of the BSI-9 were found to be of comparable model fit to that of the BSI-18, with the CFI, TLI, SRMR, and RMSEA falling within acceptable values when the items were modeled categorically rather than being treated as if they were continuous variables (Table 4). The chi-square value was significant. However, large sample size has been reported to skew this statistic [27] and the current study sample was of exceptionally magnitude with > 1000 participants per subsample. In sum, the statistical indices reflect a good model fit for the three-factor structure of the BSI-9 and an acceptable fit of the one-factor model of the BSI-9. Clinical judgment can be used to determine which model (one factor versus three factors) would be most useful for the purpose of the assessment.

**Table 4:** Confirmatory factor analysis models of the BSI-18 and BSI-9

Scale	Model	Chi-square	df	RMSEA [90% C.I.]	TLI	CFI	SRMR
BSI-18	1-factor model	1133.39	135	0.08 [0.07-0.08]	0.94	0.95	0.06
	3-factor model, correlated factors	576.26	132	0.05 [0.05-0.06]	0.97	0.98	0.04
BSI-9	1-factor model	512.41	28	0.12 [0.11-0.13]	0.92	0.94	0.06
	3-factor model, correlated factors	80.78	24	0.04 [0.03-0.06]	0.99	0.99	0.02

Note. *p* < .001 for all analyses reported; df = Degrees of Freedom; RMSEA = Root Mean Square Error of Approximation; C.I. = Confidence Interval; TLI = Tucker Lewis Index; CFI = Comparative Fit Index; SRMR = Standardized Root Mean Square Residual

Of note, the model fit for the BSI-18 reported in the present study is different from the fit reported for the development of the BSI-18 by [16], which used the same data set. The difference can be attributed to how the BSI data were modeled: current analyses fit the items as categorical data while Franke used continuous data modelling.

In both the one- and three-factor models, each item loaded strongly onto the global score and its corresponding subscale (Table 3) and had good reliability. Although Cronbach’s alpha was lower, albeitacceptable, for the subscales, this may be considered a strength rather than a limitation. Higher alpha levels for a very short subscale, such as in the BSI-9, could in fact be considered a limitation, as it may indicate too much similarity between items and therefore only measure one facet of a larger construct. We aimed to capture different indicators of each of the subscale to better encompass the construct, which led to the slightly lower alpha values. In terms of convergent validity, the correlations of anxiety, depression, and somatisation subscales and total BSI scores with anxiety, depression, and total scales of both the PHQ-4 and HSCL-25 (Table 2) were moderate to large, with somatisation showing smaller correlations with the other measures. This is not surprising, given that neither validation measure (i.e., the PHQ-4 and HSCL-25) contains a somatisation subscale, which would be expected to correlate strongly with the BSI-9 somatisation subscale. Correlations with specific subscales of the PHQ-4 and HSCL-25 were slightly smaller than expected, which may be attributed to slight measurement differences captured by the wording of scale items (i.e., different facets of the constructs are measured on different scales).

### Limitations

Although the size and representative nature of the sample used in the current study reflect a methodological strength, several limitations should be considered. First, the sample used to develop the BSI-9 was the same sample used by [16] to validate the German version of the BSI-18; ideally this would have been a separate sample. Secondly, a lack of longitudinal data prevents the evaluation of predictive validity. An additional, more recent dataset could be useful to compare the changes in distress in the German population over time and provide a more current picture of the general population’s distress levels. Third, the present study used a limited number of distress measures for convergent and divergent validity. Using additional measures, especially a scale capturing somatization symptoms would provide further insight into the validity of the BSI-9. Relatedly, the smaller correlation between subscales of measures used for convergent validity (i.e., the HSCL-25 and PHQ-4) may also capture a more clear distinction between anxiety and depression subscales compared to that measured by the BSI-9.

### Implications & future directions

The BSI-9 is a short distress screening tool for research applications and that clinicians may use with their patients. Clinicians can use their clinical judgement to determine if they would like to use the three factor or one factor structure, depending on the information they are seeking. Future investigations of the BSI-9 should examine its utility in clinical populations, as well as in other linguistic and cultural settings. Further, since psychological distress may be influenced by a variety of factors, such as age, education level, relationship satisfaction, worry about loved ones, and environmental stress and has implications for mental health, quality of life, and general well-being [35, 36], future studies should examine how differences in these demographic characteristics are related to BSI-9 scores.

Given the broad nature of distress, the BSI-9 provides a fast screening tool for clinicians to screen their patients’ distress levels. It is a valid and reliable measure that has the potential to be used in both research and clinical settings.

## Data Availability

The datasets used and/or analysed during the current study available from the corresponding author on reasonable request.
